# Design, modeling and *in silico* simulation of bacterial biosensors for detecting heavy metals in irrigation water for precision agriculture

**DOI:** 10.1016/j.heliyon.2024.e35050

**Published:** 2024-07-23

**Authors:** Francisco Salcedo-Arancibia, Martín Gutiérrez, Arturo Chavoya

**Affiliations:** a Universidad de Guadalajara, Centro Universitario de Ciencias Económico Administrativas, Departamento de Sistemas de Información, Periférico Norte No. 799, Núcleo Universitario Los Belenes, Zapopan, Jalisco, CP 45100, Mexico; b Universidad Diego Portales, Escuela de Informática y Telecomunicaciones, Ejército No. 441, Santiago, CP 837 0007, Chile

**Keywords:** Biosensors, *In silico*, Precision agriculture, Gene circuits, Heavy metal detection

## Abstract

Sensors used in precision agriculture for the detection of heavy metals in irrigation water are generally expensive and sometimes their deployment and maintenance represent a permanent investment to keep them in operation, leaving a lasting polluting footprint in the environment at the end of their lifespan. This represents an area of opportunity to design new biological devices that can replace part, or all of the sensors currently used.

In this article, a novel workflow is proposed to fully carry out the complete process of design, modeling, and simulation of reprogrammable microorganisms *in silico*. As a proof-of-concept, the workflow has been used to design three whole-cell biosensors for the detection of heavy metals in irrigation water, namely arsenic, mercury and lead. These biosensors are in compliance with the concentration limits established by the World Health Organization (WHO). The proposed workflow allows the design of a wide variety of completely *in silico* biodevices, which aids in solving problems that cannot be easily addressed with classical computing.

The workflow is based on two technologies typical of synthetic biology: the design of synthetic genetic circuits, and *in silico* synthetic engineering, which allows us to address the design of reprogrammable microorganisms using software and hardware to develop theoretical models. These models enable the behavior prediction of complex biological systems. The output of the workflow is then exported in the form of complete genomes in SBOL, GenBank and FASTA formats, enabling their subsequent *in vivo* implementation in a laboratory.

The present proposal enables professionals in the area of computer science to collaborate in biotechnological processes from a theoretical perspective previously or complementary to a design process carried out directly in the laboratory by molecular biologists. Therefore, key results pertaining to this work include the fully *in silico* workflow that leads to designs that can be tested in the lab *in vitro* or *in vivo*, and a proof-of-concept of how the workflow generates synthetic circuits in the form of three whole-cell heavy metal biosensors that were designed, modeled and simulated using the workflow. The simulations carried out show realistic spatial distributions of biosensors reacting to different concentrations (zero, low and threshold level) of heavy metal presence and at different growth phases (stationary and exponential) that are backed up by the whole design and modeling phases of the workflow.

## Highlights

• Genetic circuit designs can be made *in silico* before their actual implementation.
• Genetic circuits can be used as detectors for heavy metals in irrigation water.
• Strains of *Escherichia coli* can be used as biodetectors through genetic engineering.
• A workflow is proposed to design, model and simulate genetic circuits *in silico*.

## 1 Introduction

The official definition of precision agriculture (PA) approved in 2024 by the International Society of Precision Agriculture (ISPA) states that it is a management strategy that retrieves, processes and analyzes spatiotemporal data, combining them with complementary information, enabling informed and timely decision making concerning crops to improve resource use, productivity, quality, rentability and sustainability of agricultural production [1]. Spatiotemporal analysis and control carried out in PA consists of studying differences in fertility and growth of crops in distinct sections of fields, and the analysis of production in a single given field in different seasons.

A central part in PA is the use of information technologies (IT), as it improves agricultural processes, maximizing production and reducing investment costs and environmental impact [2,3]. To assess soil fertility, crop development (both related to spatial variation) and harvest development (referred to temporal variation), PA uses a large array of technological devices. Concretely, synthetic biology technology used for PA leans on five different technologies [[4], [5], [6], [7]]. These technologies are based on given components, such as genes, proteins, and cell parts, among others. They give rise to computation needed to determine, for instance, the presence or absence of heavy metals in water [[4], [5], [6], [7], [8]]. These metals can make their way into hydric systems from different sources, such as casting and refinement processes, fuel consumption, chemical element leaks, and sewage water discharge [9]. One part of a solution to sensing the presence of these metals involves microorganisms that act as living computers based on gene circuits encoded with DNA [10] that process sensing information and control their biological functions [11,12].

Unlike classic computing capabilities that are constrained by structural features and electronic components, cellular consortia exhibit a more flexible and reconfigurable architecture. This allows the cell population to change and dynamically adjust its physical composition and interactions to adapt on the fly in solving a problem in the most efficient manner [13]. Individual reprogrammed cells are limited by the actions that a single cell is capable of carrying out. Therefore, to tackle more complex problems, a multicellular approach is preferred, as it can distribute processing among multiple cells [14,15]. The design of synthetic biocircuits within innocuous strains of *Escherichia coli* (*E. coli*) bacteria emerges as an alternative to current sensors used for PA [16]. The proposed bacterial biosensors represent a less environmentally aggressive option, as they do not require any hardware or software for their operation. They utilize chromatic proteins from reporter genes, enabling the visual detection of heavy metals in water, making the results easily perceptible at a glance. A modular design of such circuits is achieved by reusing standard biological parts that exhibit a transcriptional response. Chromatic proteins are used as reporters as they have some advantages over fluorescent proteins, such as producing dark colors within environmental lighting, allowing for a cheaper analysis without lab instruments. Therefore, biosensor experimentation results can be promptly viewed [17,18].

Irrigation water is a vital element necessary to many processes involving food production. This research work focuses on the assessment of irrigation water quality for precision agriculture by determining the presence or absence of hazardous amounts of heavy metals. This work contributes to advancing the sensor dimension by proposing whole-cell sensors that detect hazardous amounts of heavy metals in irrigation water. Some of those metals, such as lead, mercury, arsenic, chrome, aluminum, and zinc, are very toxic even at low concentrations [[19], [20], [21], [22], [23]]. When these metals transfer from water to other elements such as plants, animals or humans, a bioaccumulation of the metals can occur in the organism. Such contaminants can be fatal to human beings, as accumulation of heavy metals are concentrated in organs such as the heart, kidneys and lungs [24]. To tackle the problem of detecting heavy metals from an IT perspective, the design, modeling and simulation of living visual reporting devices were carried out in the present work by using *in silico* synthetic biology and other IT tools. The proposed devices are an alternative to electronic or hybrid sensors that are currently being used for the same goal in PA. Bacterial biosensors offer several advantages over conventional sensors in PA, including higher sensitivity and specificity, lower production costs, sustainability and biodegradability, self-replication capability, and real-time *in situ* measurement. Additionally, they allow for the simultaneous detection of multiple parameters, can be genetically adapted to specific conditions, and integrate well with synthetic biology technologies. These features make them an efficient and sustainable tool for agricultural monitoring and management. Specifically, three synthetic gene circuits were designed *in silico* for innocuous strains of *E. coli* that detect arsenic, mercury or lead in irrigation water. The outcome of the developed pipeline are data that relate to spatial variation in PA, specifically soil fertility and assessment of physicochemical factors, mainly referred to the presence of heavy metals in irrigation water under different conditions. To implement the solution with IT tools, three software platforms were prioritized to cover the design, modeling and simulation (*iBioSim*, *TinkerCell* and *gro*, respectively). Additionally, two online biological standard parts repositories (*iGEM BioBricks* and *SynBioHub*) [[25], [26], [27], [28], [29]] were used to validate and preserve coherence of all involved biological elements. These applications and online platforms ensured an *in silico* process that complies with the international synthetic biology standard SBOL. This fact helps in the replication of experiments in other platforms and their future implementation *in vivo* in a molecular biology laboratory.

## 2 Materials and methods

From an IT perspective, it is very useful to have the appropriate software for solving the problems at hand. Some of these applications could even be essential. Validation across different platforms strengthens the proposed designs and adds robustness. For this work, two repositories of standard biological parts and three software platforms were used during the development stages. After the selection of the biological parts forming each circuit as *BioBricks*, a biocompatibility validation of said biological parts was performed using the *SynBioHub* platform. This cross-validation between *BioBricks* and *SynBioHub* allowed for an optimal selection of the elements for designing each circuit. All of the mentioned repositories and tools in the development pipeline are briefly specified below.

• **Registry of Standard Biological Parts:** The *iGEM* repository includes parts (*BioBricks*) for assembling synthetic gene circuits. This repository stores more than 20,000 documented biological parts [30,31].
• ***SynBioHub*:** A repository for scientists that design biological constructs. It allows for uploading DNA designs and proteins and assesses their compatibility. *SynBioHub* also aids in the browsing and retrieval of information on useful existing parts and designs by combining a large variety of sources [29].
• ***iBioSim v3.0*:** A design and simulation tool centered on gene circuits specified in SBML or SBOL v3.0 formats [32]. It can analyze metabolic networks and cellular signal pathways, but also allows for modeling and visualization of multicellular and spatial models [33].
• ***TinkerCell v1.2.876*:** Includes a sufficiently detailed diagram for mapping models or experimental results. Furthermore, multiple mathematical analyses on those models can be executed along with single-cell simulations [31,[34], [35], [36]].
• ***gro v. beta.6*:** A language for programming, modeling, specifying and simulating cell behavior in growing microcolonies. It can simulate cell growth, division, noise, and molecular signal diffusion [14,27].

**Fig. 1:**
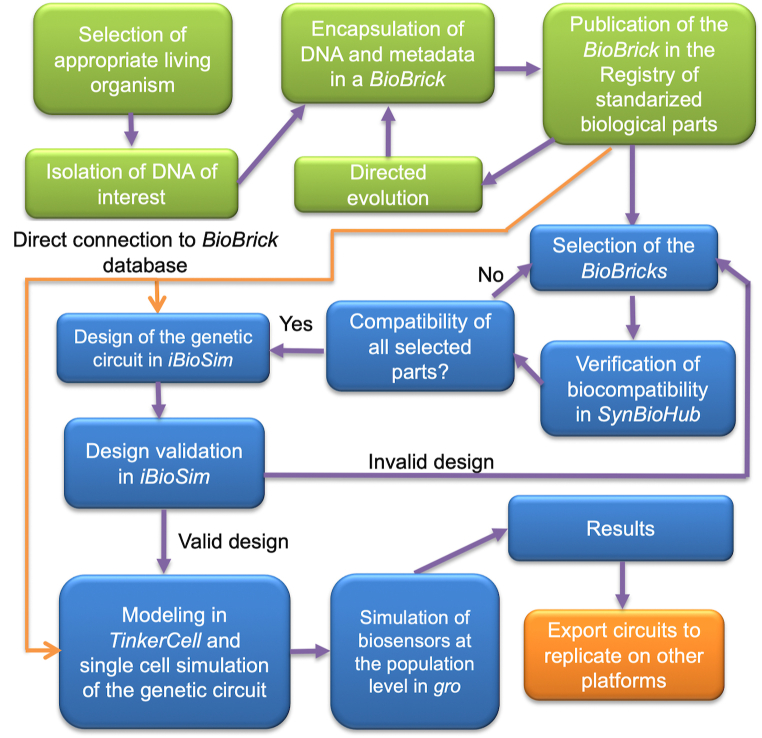
Heavy metal bacterial biosensor development methodology. Green colored boxes refer to work carried out by geneticists, blue boxes are activities covered by the currently presented development pipeline, whereas orange boxes denote the external interface and derived activities that will allow for the project to continue by being implemented either in other platforms or *in vivo* in a wet lab [37]. (For interpretation of the references to color in this figure legend, the reader is referred to the Web version of this article.)

The final step of the development pipeline was to export the whole design of each circuit to SBOL, FASTA and GenBank formats. The standardization of data into these formats allows its use by a wide range of synthetic biology practitioners, ranging from end-users to software developers, as well as wet lab biologists who can replicate and implement such circuits *in vivo* at any lab that uses the mentioned standards. Fig. 1 summarizes the methodology that we followed for all of the presented development pipeline.

The proposed methodology was conceived to develop any kind of living reprogrammed device and to enable its *in vivo* implementation as the outcome of the development pipeline. Such outcome is then exported to major synthetic biology standard formats. The development method also allows the use of different software applications from the ones used in this project for the design, modeling, and simulation phases. Thus, the presented methodology is flexible and adaptable to a variety of synthetic biology projects.

## 3 Results

The workflow that will be described in this section allows the exchange of standardized biological parts, choosing the most appropriate specialized software for each problem to be solved for the different phases of the process. In addition, it is possible to export the results of the entire process developed *in silico* to SBOL, GenBank and FASTA standards. This would allow its subsequent prompt implementation *in vivo* or *in vitro* in a molecular biology wet lab setting, going from a theoretical proposal to an experimental one.

Only the biosensor for detecting arsenic will be fully described, as the design, modeling and simulation processes for the other two biosensors are analogous. The only difference is in the selection of the *BioBricks* responsible for dimerizing the target heavy metal, the promoter sensitive to the target heavy metal, and the reporting chromatic protein for each of the sensors. In spite of only highlighting the design, modeling and simulation of the arsenic biosensor, results for all of the biosensors are shown later in this document (Table 3, Table 4, Table 5, Table 6).

**Table 1:**
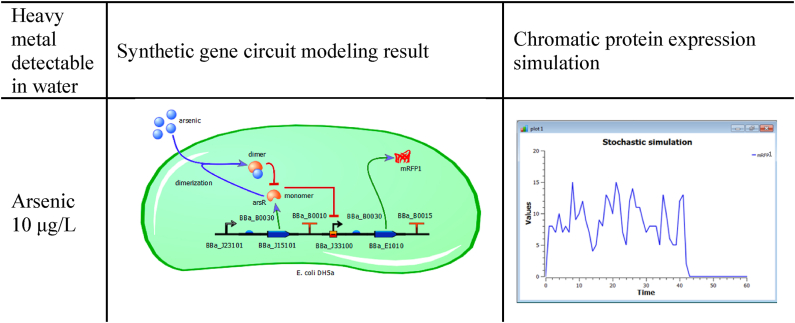
Modeling of synthetic gene circuit for arsenic detection performed with *TinkerCell*. The “Values” axis in the output graph stands for the number of synthetic gene circuit copies that are expressing the reporter chromatic protein, whereas the “Time” axis units are in minutes.

**Table 2:** List of parameters and values used in the arsenic, mercury and lead bacterial biosensor population-level simulations with *gro*.

Parameter	Value
Arsenic biosensor operons	arsR and mRFP1
Mercury biosensor operons	merR and amilCP
Lead biosensor operons	pbrR and amilGFP
Arsenic environmental concentration	10 μg/L, 5 μg/L and 0 μg/L
Mercury environmental concentration	6 μg/L, 3 μg/L and 0 μg/L
Lead environmental concentration	10 μg/L, 5 μg/L and 0 μg/L
Arsenic biosensor activation threshold	10 μg/L
Mercury biosensor activation threshold	6 μg/L
Lead biosensor activation threshold	10 μg/L
Growing state bacterial population count	Initial: 1000 bacteria; final: 20,000 bacteria
Stationary state bacterial population count	2000 bacteria fully immersed in the respective solution
Induced error rate	0.1 % at each simulation step

**Table 3:**
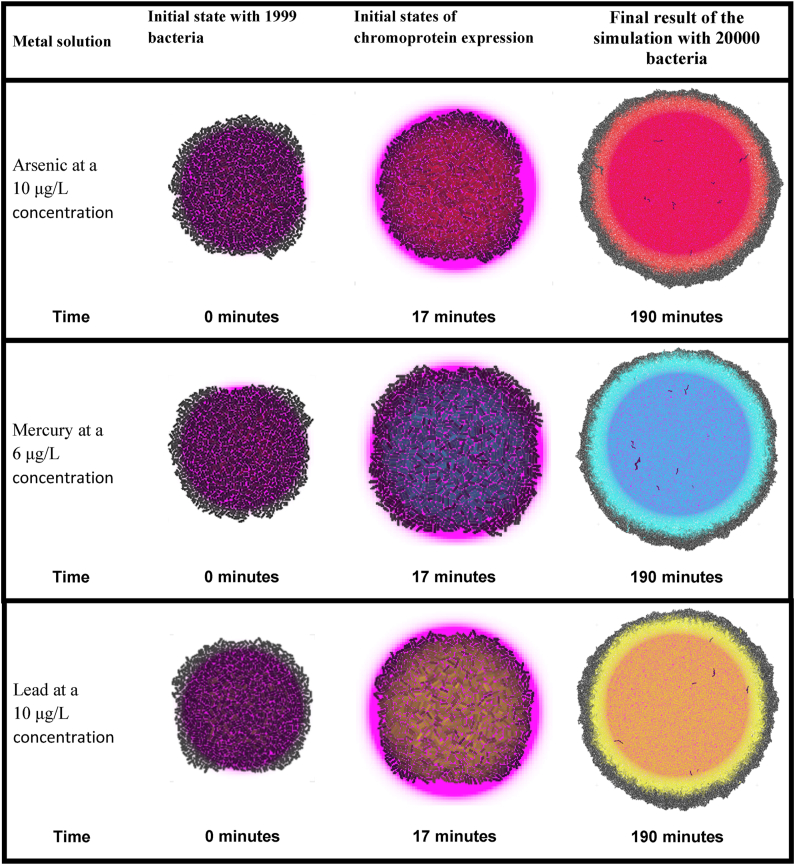
Result of the growth population simulation of the bacterial biosensors that detect heavy metals in *gro* by pouring the solution with the heavy metal in the center. In order to have a visual perception of the dimension of the biosensors, the population size was limited to a maximum of 20,000 bacteria.

**Table 4:**
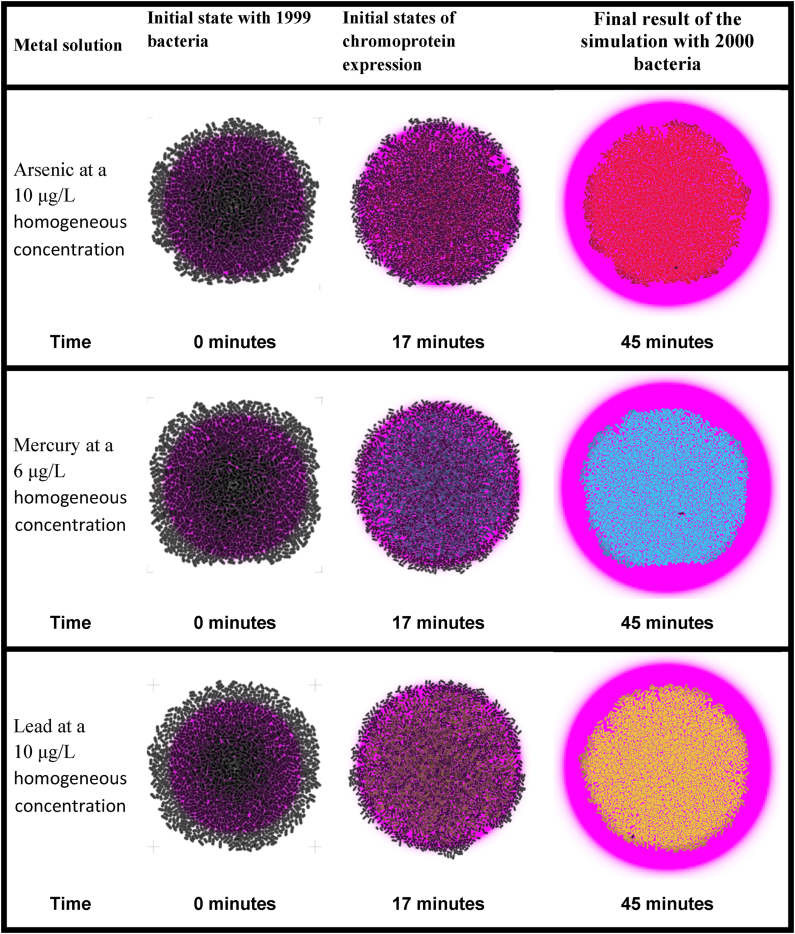
Result of the steady-state population simulation of the bacterial biosensors in *gro* completely immersed in a solution of the heavy metal corresponding to a concentration equal to the activation threshold. In this set of simulations, the bacterial population was limited to 2000 with the intention that all of them were completely immersed in the solution with the heavy metal evaluated.

**Table 5:**
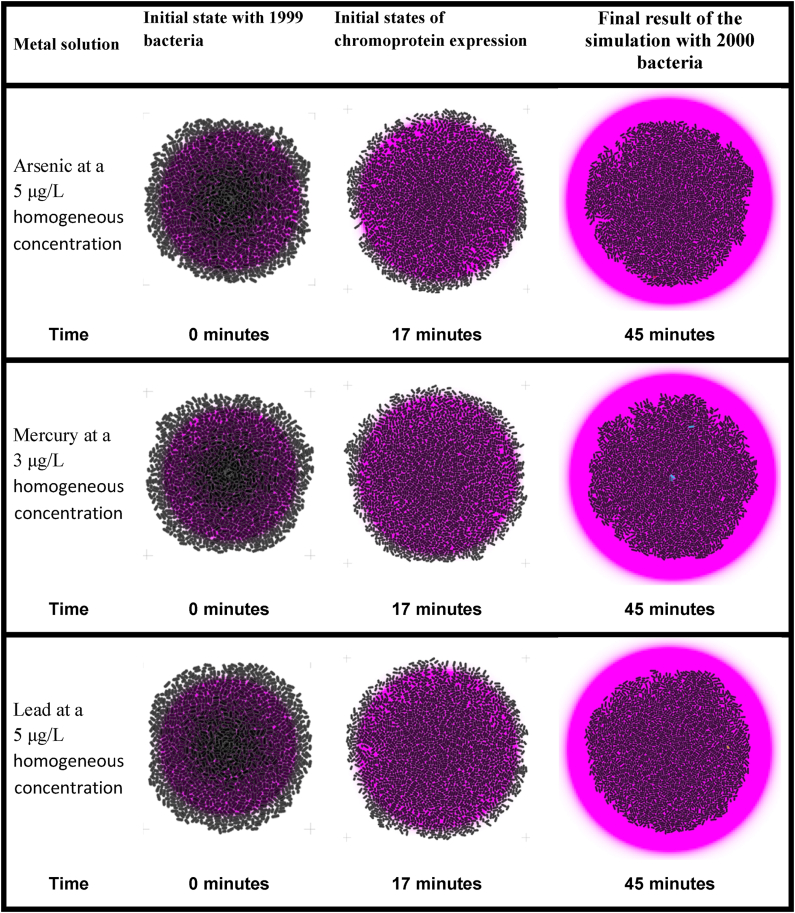
Result of the steady-state population simulation of the bacterial biosensors in *gro* completely immersed in a solution of the corresponding heavy metal with concentrations lower than the activation threshold. In order to have a convenient visual perception of the dimension of the biosensors, the population size was limited to a maximum of 2000 bacteria in a steady state (without population growth).

**Table 6:**
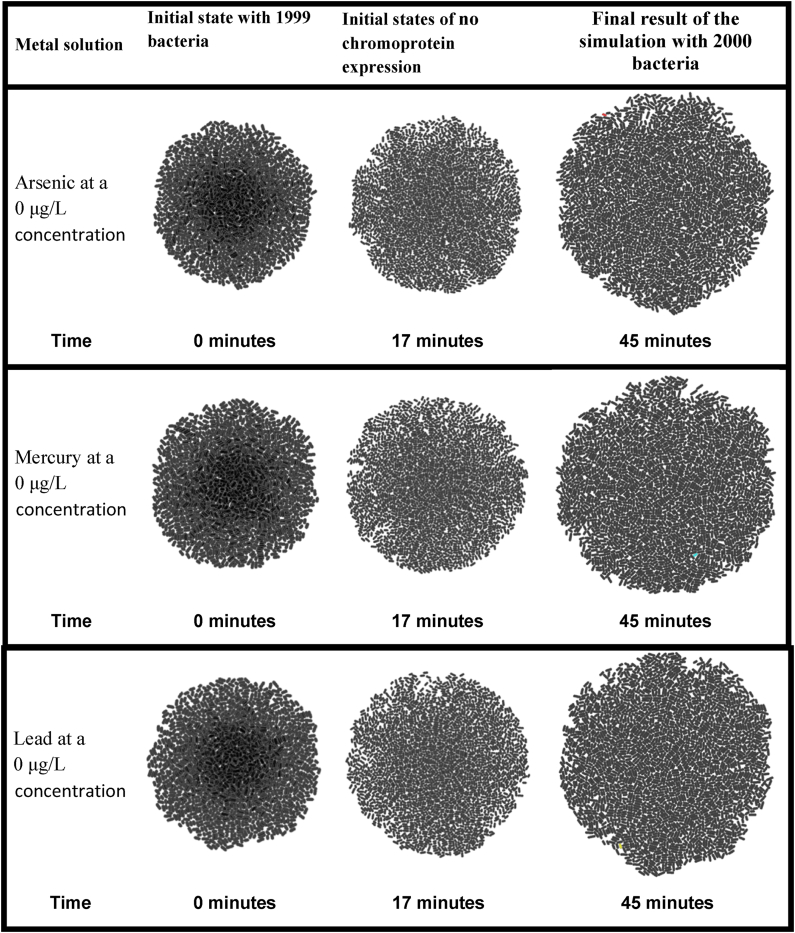
Result of the steady-state population simulation of the bacterial biosensors in *gro* completely immersed in water without any metal. In order to have a visual perception of the dimension of the biosensors, the population size was limited to a maximum of 2000 bacteria in a steady state (without population growth).

### 3.1 Arsenic detector gene circuit design

The gene circuit was designed with the *iBioSim 3* software and connected to the *BioBricks* and *SynBioHub* repositories. This design was composed by selecting eight biological parts for its construction.

The following *BioBricks* were selected from the *iGEM* standardized biological parts repository to design the arsenic detecting gene circuit in a tandem manner:

1) BBa_J23101: Constitutive promoter
2) BBa_B0030: Ribosome Binding Site (RBS)
3) BBa_J15101: arsR gene coding region
4) BBa_B0010: Terminator
5) BBa_J33100: Arsenic sensitive promoter
6) BBa_B0030: RBS
7) BBa_E1010: mRFP1 gene coding region
8) BBa_B0015: Bidirectional double terminator

**Fig. 2:**
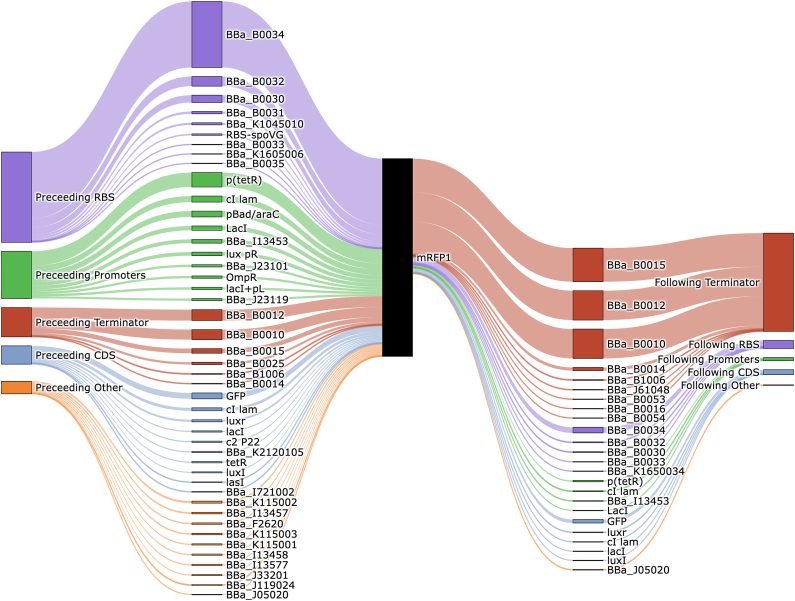
Sankey diagram of *BioBrick* BBa_E1010 or mRFP1 obtained from the *SynBioHub* repository. The central black block represents the *BioBrick* to be used. On the left and right sides, the most commonly used predecessor and successor *BioBricks* with mRFP1 are visualized. The thickness of the lines indicates the number of publications that have utilized each *BioBrick* in connection with the analyzed *BioBrick* (mRFP1).

Once the selection of the biological parts was done, *SynBioHub* was used to test the coherence of the design. The biocompatibility of the parts used can be validated with the Sankey diagram, as illustrated in Fig. 2.

**Fig. 3:**
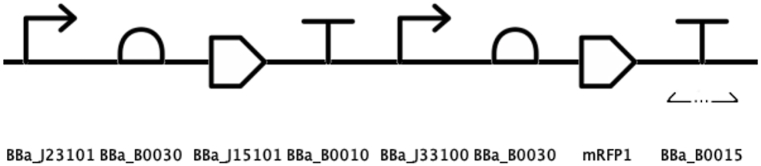
Arsenic detector gene circuit designed with *iBioSim*. In this tool, the BBa_E1010 *BioBrick* is represented by its chromoprotein product mRFP1.

Then, each of the parts was retrieved from the *BioBricks* repository to be used in the *iBioSim* software and build the arsenic biosensor circuit design shown in Fig. 3.

The *BioBricks* used in the circuit design are briefly described next. The BBa_J23101 *BioBrick* is a constitutive promoter for the whole circuit. It regulates *BioBricks* BBa_B0030 and BBa_J15101 (the RBS and the arsR coding region, respectively). The circuit then ends with the BBa_B0010 *BioBrick* (sequence terminator). The arsR protein acts as follows: when arsenic is absent from the environment, arsR represses promoter BBa_J33100, inhibiting the expression of the red chromatic protein mRFP1 (*BioBrick* BBa_E1010), but when arsenic is present, arsR dimerizes with this metal, removing the repression on the arsenic sensitive promoter and allowing for the reporting protein to be expressed and be visible to the human eye. Transcription of the circuit then ceases thanks to the bidirectional double terminator BBa_B0015.

Mercury and lead biosensors were designed in an analogous manner as described for the arsenic sensor, but replacing *BioBricks* 3, 5 and 7 used in the assembly with the following *BioBricks*:

Mercury biosensor – *BioBrick* 3 was replaced with BBa_K1420004 (merR), *BioBrick* 5 was replaced with BBa_K356002 (mercury sensitive promoter), and *BioBrick* 7 was replaced with BBa_K592009 (blue chromatic protein) in the arsenic biosensor assembly sequence.

Lead biosensor – *BioBrick* 3 was replaced with BBa_I721002 (pbrR), *BioBrick* 5 with BBa_I721001 (lead sensitive promoter), and *BioBrick* 7 with BBa_K592010 (yellow chromatic protein) in the arsenic biosensor assembly sequence.

Once all gene circuits were designed and validated, their respective metabolic network was described in *TinkerCell*. Taking into account the previously shown similarities among the circuits, the text will proceed only with the modeling and simulation of the arsenic biosensor.

### 3.2 Modeling the metabolic network of the arsenic detector gene circuit with *TinkerCell*

*TinkerCell* is a computer-assisted design (CAD) tool for synthetic biology. It is a software that simulates genetic circuits constructed using standard parts running inside a single cell. *TinkerCell* is designed under the assumption that the future of synthetic biology will be an intricate interaction between a large array of experimental techniques, databases storing experiment results, and mathematical models that explain different aspects of them. Unfortunately, *TinkerCell*, is no longer under active development, which entails many limitations for such tool, but is still useful for the purposes of this study, as mathematical modules for modeling enzyme kinetics are robust and visually more illustrative than other synthetic biology applications. Thus, *TinkerCell* is a valuable tool for simulating single cell dynamics, and is currently still used, as reported in recent works [31,34,38].

Data related to each *BioBrick* was retrieved from the *iGEM* database using the WikyDust software, which is embedded in *TinkerCell*. The predefined mathematical models of the software were used to carry out the modeling process. This allowed to simulate the behavior of all three heavy metal biosensors at a single-cell level.

For the arsenic biosensor, the *BioBricks* and their function are described below:

1. Constitutive promoter (BBa_J23101): A promoter is composed of a DNA sequence that binds to the RNA polymerase (RNAP). BBa_J23101 is a constitutive promoter, meaning that it constantly transcribes DNA into messenger RNA (mRNA) without the need for any external factor to trigger it.
2. Ribosome Binding Site (RBS) (BBa_B0030): It is a sequence that when transcribed into mRNA catalyzes the translation process.
3. Coding Sequence (CDS) (BBa_J15101): This *BioBrick* gene translates into the arsR protein, which is responsible for repressing the BBa_J33100 *BioBrick* in absence of arsenic in the environment. This repression, in turn, inhibits the production of the red chromatic protein (described below). When arsenic is present in the environment, the arsR protein binds to arsenic molecules forming a dimer that neutralizes the repression that the arsR monomer exerted on the arsenic sensitive promoter. This allows for the red chromatic protein gene (BBa_E1010) to initiate its expression.
4. Terminator (BBa_B0010): Interrupts the transcription process by dissociating the RNAP from the mRNA.
5. Repressible promoter (BBa_J33100): While the arsR monomer is present, this promoter stays repressed (therefore no reporter chromatic protein is produced). When arsR dimerizes with the arsenic present in the environment, repression on the arsenic sensitive promoter is neutralized and the transcription process is triggered. Then, the translation process completes the production of the red chromatic protein.
6. Ribosome Binding Site (RBS) (BBa_B0030): This is the same *BioBrick* as in (2) above.
7. Coding Sequence (CDS) (BBa_E1010): Upon detection of arsenic in the environment and the neutralization of the arsR repression, this gene translates into the red reporter chromatic protein mRFP1, which can be readily visible by the human eye without the need for additional hardware.
8. Bidirectional double terminator (BBa_B0015): This sequence stops transcription of the whole circuit in both directions.

**Fig. 4:**
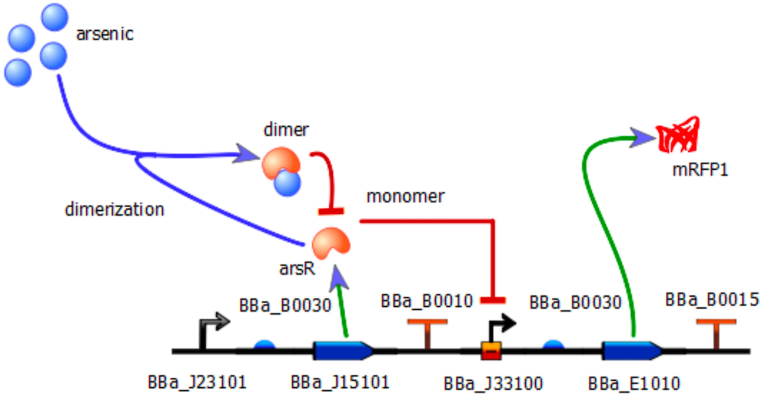
Metabolic network of the arsenic detector gene circuit modeled with *TinkerCell*. As a complement to the design of the gene circuit carried out with *iBioSim*, *TinkerCell* assisted in defining metabolic networks in which each of the *BioBricks* in the circuit interacts. It describes the repression process, dimerization, and reporter chromatic protein expression in a more precise manner for each of the proposed gene circuits.

Modeling of the full arsenic biosensor metabolic network is summarized in Fig. 4.

Metabolic network modeling for each of the gene circuits in *TinkerCell* allowed for the validation of their correct operation at a single-cell level by simulating the expression of the reporter chromatic protein. Table 1 shows a summary of the modeling results for the arsenic biosensor.

After describing the metabolic network of the arsenic detecting gene circuit, it was implemented in a simulated *E. coli DH5α*, which sheds light on the operation of the circuit at a single-cell level. As additional parameters for the mathematical modules of *TinkerCell*, 15 copies of the circuit were used within the bacterium and a homogeneous concentration of 10 μg/L of arsenic was present in the environment.

Modeling results from *TinkerCell* not only validated the coherence of the design built with *iBioSim*, but also helped to observe each circuit function when the minimum threshold concentrations were present: 10 μg/L for arsenic, 6 μg/L for mercury, and 10 μg/L for lead. The graphical output of the simulation for the detection of arsenic at the single-cell level is shown in the third column of Table 1. The *x*-axis of the graph shows response time in minutes, whereas the *y*-axis represents the number of gene circuit copies expressing the reporter chromatic protein.

The output graphs show variability in the number of copies of the circuit expressing the reporter chromatic protein over time. This variability is dependent on the chosen parameters. Therefore, to complement *TinkerCell* results, population-level simulations were carried out. This allowed for a closer characterization of the effect of the environment on biosensor populations. Multi-cell simulations were performed using the *gro* software.

### 3.3 Arsenic detecting biosensor simulation with *gro*

The *gro* software uses a programming environment implemented in C++ and simulates multicellular gene circuits in 2D. Its graphical user interface (GUI) displays bacterial population growth and cell-to-cell communication, among other features. The *gro* simulations are coded in a specification language called *gro*. Once all parameters and functionalities are set in the code, the software simulates multi-cell behaviors over time, such as colony growth, cell division, intrinsic and extrinsic noise, molecular interactions, and reporter protein expression, among other cellular functions [27]. The *gro* software is useful for synthetic biology as distributed multicellular behaviors can be prototyped, but also for assessing that specified interaction rules produce the desired global result. The language specifies behavior at different abstraction levels, ranging from high-level code to low-level biomolecular interactions [27].

In the simulations with *gro*, two different conditions were tested: 1) growing state and 2) stationary state. In the growing state, bacteria reproduce while at the same time a solution with a fixed concentration of the tested heavy metal was continuously poured in the center of the colony and allowed to diffuse towards the periphery. The purpose of this condition was to simulate the reaction of the biosensors when the metal was present at different concentrations along the radial axis due to the diffusion of the solution towards the boundary of the colony. In the stationary state, bacteria had virtually no reproduction and were completely submerged in a solution with the tested heavy metal at a determined concentration. The purpose of this simulation was to predict real-world use of the biosensors when tested with a sample of the evaluated irrigation water.

**Fig. 5:**
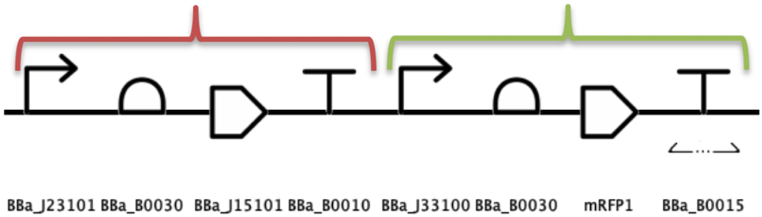
The arsenic detecting circuit was assembled in the form of two operons to configure it for a *gro* simulation. The operons were named arsR (red bracket) and mRFP1 (green bracket). (For interpretation of the references to color in this figure legend, the reader is referred to the Web version of this article.)

The first step in configuring the required parameters for the *gro* simulation was to separate the gene circuit in two operons: arsR and mRFP1. The first operon contains the *BioBricks* involved in the production of the repressor protein and arsenic transporter: constitutive promoter, RBS, CDS and sequence terminator. The second operon groups *BioBricks* that detect arsenic level and express the red reporter chromatic protein: repressible promoter, RBS, CDS and double transcriptional terminator. These operons are depicted in Fig. 5.

The second step in adapting the code for a *gro* simulation is to define the necessary parameters to carry out simulations that best approximate the deployment of biosensors in a controlled external environment. For instance, a Petri dish with different concentrations of the respective heavy metal can be simulated. Such parameters are shown in Table 2.

**Fig. 6:**
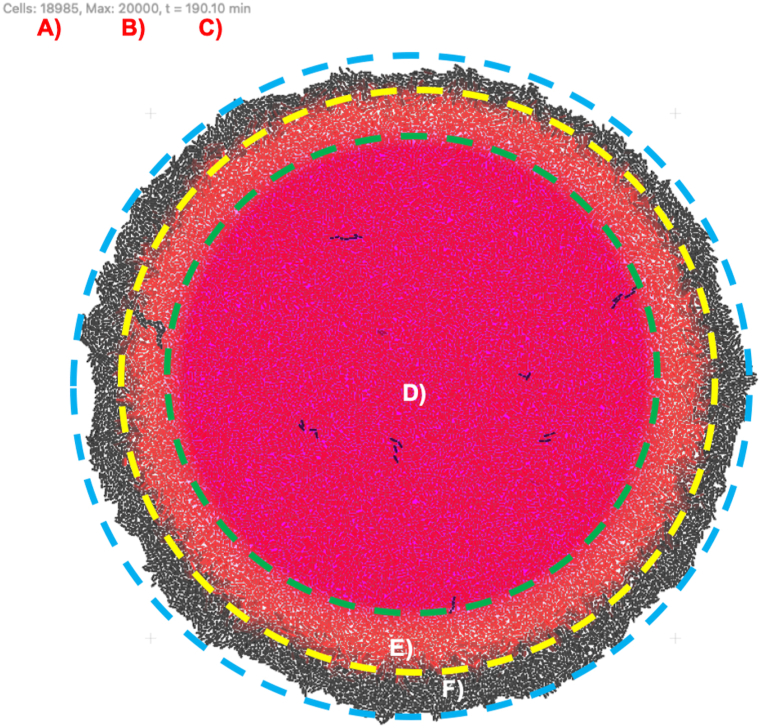
Execution of a simulation of arsenic biosensors in *gro*: A) Real-time bacteria count. B) Maximum population. C) Elapsed time. D) Initial area with arsenic, which over time diffuses to the boundary marked by E). E) Active biosensors in response to arsenic, covering D) and E). F) Inactive biosensors outside the reach of arsenic.

The third step in relation to the arsenic detecting bacterial biosensors deployment was to configure the *gro* source code to test all values mentioned in Table 2. Once the source code for each of the biosensors was written, a series of four population-level simulations were executed in *gro*. One of them was conducted in a growth state with activation thresholds, and the other three in a steady state: one with heavy metal solutions at the activation threshold, another with thresholds below the activation level, and the last one in heavy metal-free solutions. The result of one of these population-level simulations in growing state is shown in Fig. 6.

In the population-level growing state simulation of the arsenic detecting biosensor shown in Fig. 6, a total of 18,985 living cells were present at time 190.10 min, from a maximum of 20,000 possible cells for the simulation. The magenta background represents the arsenic at a homogeneous central concentration of 10 μg/L. The red cells report the presence of arsenic in sufficient concentration in their respective zone, whereas black cells are inactive. This can happen either because they are out of the range of the arsenic concentration at the threshold level, or due to a malfunction in the circuit (a parameter of the simulation).

In order to have a comparative baseline for the simulated growth of the proposed bacterial biosensors, for each metal it was decided to run a series of simulations in a stationary state—i.e. with virtually no bacterial growth—starting with 1999 bacteria and with a maximum population of 2000 bacteria immersed in a solution with a constant metal concentration.

The enzyme kinetics parameters used for these simulations in stationary state were the same as those used in the simulations with the growing bacterial populations. The only difference being the growth rate, which was modified to such a low value that it allowed simulating the complete activation process of the whole population of biosensors prior to the end of the simulation. In the stationary state, variations in the concentration of the heavy metals were tested to corroborate the correct functioning of the biosensors under different scenarios.

Table 3 presents the results of the simulation of the three bacterial biosensors at the population level. Each biosensor is organized in rows according to the concentrations of the heavy metal at their respective activation thresholds. The arsenic biosensor expresses the red chromoprotein, the mercury biosensor expresses the blue one, and the lead biosensor expresses the yellow one. In the columns, different moments of the simulation of the growing biosensors at the population level are shown.

As can be observed, the bacteria that are outside the range of the heavy metal due to its dilution do not express the corresponding chromoprotein. In a similar manner, the bacterial biosensors that are within the range of the heavy metal solution and do not express the chromoprotein, do so due to the induced error mentioned in Table 2. This was done to obtain a more realistic approximation of what an *in vitro* or *in vivo* deployment would be like.

Subsequently, three series of simulations were performed for each of the biosensors at the population level, all in steady state, to obtain a more accurate visual perspective of their functionality.

Table 4 shows the temporal progress of the execution of the steady-state population-level simulations of the three proposed biosensors in the columns. In the rows, each of the three biosensors is presented, fully immersed in a homogeneous solution with concentrations at the activation threshold of the heavy metal to be detected.

As part of the set of simulations used to verify the functionality at the population level of the proposed biosensors under different conditions, Table 5 presents the results of a series of steady-state simulations with homogeneous concentrations of the heavy metal to be evaluated below the activation threshold. Following the format of the previous tables, each of the three biosensors is shown in the rows and different moments of the execution over time are presented in the columns.

Finally, Table 6 shows the initial, intermediate, and final stages of the steady-state population-level simulations for each of the proposed biosensors when the heavy metal was removed altogether from the solution in which the bacteria were immersed.

**Fig. 7:**
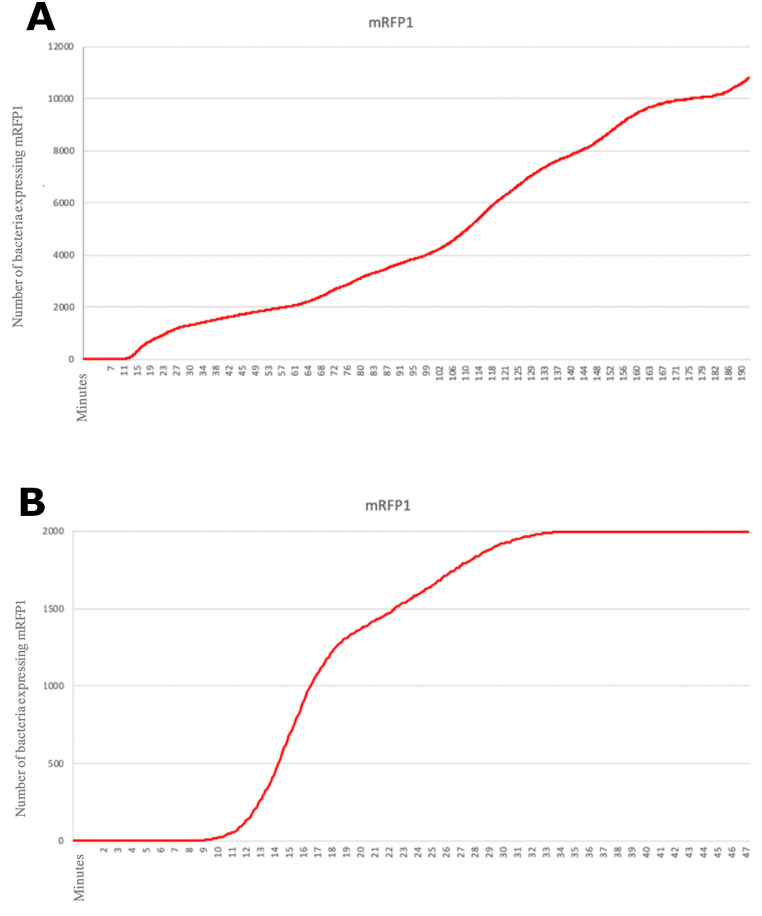
A) Graph of the number of bacteria expressing the chromoprotein over time in a growing-state simulation in *gro*. The initial and final number of bacteria were 1000 and 20,000, respectively. A solution with an arsenic concentration of 10 μg/L was continuously poured in the center of the colony. B) Graph of the number of bacteria expressing the chromoprotein over time in a stationary-state simulation in *gro*. The corresponding initial and final numbers of cells were 1999 and 2000, which were immersed in a solution with arsenic at a 10 μg/L homogeneous concentration.

Once the simulations in *gro* were completed, the corresponding graphs were generated from the data obtained and stored in CSV (comma-separated values) files. The output graphs of the simulations in the growing state and the stationary state are respectively shown in Fig. 7.

The graph shown in Fig. 7B obtained from *gro* simulations provides information regarding the number of bacteria that are expressing the corresponding chromoprotein at a population level as a function of time. For this reason, in the stationary state simulation almost the entire bacterial population is expressing the chromoprotein at the end when the metal solution is at the activation threshold. This is due to the fact that at all times the bacteria are completely submerged in the heavy metal solution. In contrast, in the growing state simulation such as that of Fig. 7A, a large number of bacteria do not express the chromoprotein. This happens because the solution with the metal is poured in the center of the colony and the metal gets diluted as it diffuses outwards of the growing bacterial population. The colony keeps continuously expanding, causing many biosensors to not reach the required activation level to detect the metal.

On the other hand, Table 5 shows that almost no biosensors express reporter proteins when the metal concentration is below the activation threshold. The few that do express the reporter protein, do so due to a random failure in the biosensor genetic circuit, which is introduced as a parameter in the simulations in *gro* to have a more realistic approximation to the use of the biosensors in a physical implementation.

It should be noted that in a real-world *in vitro/in vivo* implementation, three solutions would be added to the biosensors (in separate compartments): (1) the water sample to be tested; (2) a solution with a known concentration of the metal—for instance, at or above the activation threshold—to verify that the bacteria are viable and can detect the metal, ruling out false negatives; and (3) a solution without the metal, to make a comparison with the result from the water sample and to rule out false positives.

The proposed process of design, modeling and simulation of the bacterial circuits in the present project can be of use for researchers in related fields—such as bioremediation or pathogen identification diagnostics—due to the integration of software and online platforms that allow validation of complete consistency of the process. The proposed development pipeline could be considered as a previous step before proceeding to the implementation of the biological circuit, since the number of validations, tests and simulations carried out *in silico* allows minimizing possible design errors. It also reduces costs derived from potential inconsistencies when implementing a design directly in a molecular biology wet lab. On the other hand, the use of chromatic reporter proteins in the proposed biosensors offers the possibility of obtaining immediate visual feedback in a short period of time without the need of specialized hardware, which positions the present proposal of bacterial biosensors for detecting arsenic, mercury, and lead as a viable alternative to other methods.

All design (*iBioSim*), modeling (*TinkerCell*), and simulation (*gro*) files with complete genomes of each synthetic genetic circuit in FASTA and GenBank formats, as well as CSV files containing numerical results from various simulations, are available on GitHub at: https://github.com/fsarancibia/Heavy-Metal-BioSensors. Additionally, high-definition and time-lapse versions of all 24 simulation videos are available on YouTube in the following playlist:

https://www.youtube.com/playlist?list=PLVdaEoc79TKrFLSQWNbgDurNB2PU6eLN4.

## 4 Discussion

From an engineering point of view, it is essential to work with biological elements that have been validated and verified in the laboratory by molecular biologists. A certainty that these biological parts encapsulated in *BioBricks* have been designed and tested in actual laboratories prior to their use *in silico* is of paramount importance. On the other hand, this dependence on the availability and variety of these biological components can impose a restriction for synthetic biologists on the kind of designs they can elaborate. Regarding the repositories used for the validation of the biological parts, the *BioBricks* and *SynBioHub* platforms offer the possibility to access detailed information on each individual component. Either of these two platforms provides sufficient information on the components; nevertheless, we decided to use both platforms to have a double validation and thus reach a greater certainty on the compatibility of each *BioBrick*, as well as a guarantee for the complete consistency of each of the circuits.

It should be noted that, regarding the design process, we decided to use the international SBOL standard and also software complying with this standard—in the case of *iBioSim*—as the problem was approached from a computer science point of view and was not regarded exclusively as a biological problem. Researchers having a molecular biology background might be tempted to avoid the use of design, modeling, and simulation software, as well as the standardized SBOL symbology. Even the omission of the verification in the *BioBricks* and *SynBioHub* databases might occur, by going directly to laboratory experimentation to design a genetic system. This more empirical approach would evidently require a complete and very specialized knowledge of each of the biological components to be used. In fact, it does not consider the accumulated knowledge base that the scientific community has continuously contributed to the mentioned digital repositories. This becomes a limitation in the efficiency of such designs, as well as representing a potential higher cost derived from the adaptations caused by possible errors when directly implementing the designs in the wet lab.

For the above reasons, the present work becomes relevant from a methodological point of view, since it was conceived and designed in a modular manner, following international standards, and with an ample variety of available software options with connection to databases or digital repositories that are frequently updated. The present proposal yields designs of living devices that are validated and supported by simulated results that allow a more efficient physical implementation, with less design errors and lower costs.

Each of the proposed biosensor designs in this study was conceived from the beginning with a modular approach. This allows to adjust the activation threshold for each of them, depending on all of the corresponding *BioBrick* promoters sensitive to each metal. Such variations are carried out and tested in a wet lab and the different *BioBricks* are later integrated into the standardized biological parts database, along with the genetic sequence, metadata, and corresponding complete documentation. In principle, it is possible to adjust the sensitivity of the proposed biosensors based on the available variations of promoters, offering a wide variety of activation thresholds and parameters for different applications.

On the other hand, the simulation process with *gro* resulted in an important visual complement to exemplify the possible results in a real-world environment. With this software it was possible to perform simulations of the biosensors for arsenic, mercury, and lead in microcolonies of bacteria in two modalities of particular interest for the present project: growing state and stationary state. In the former case, simulations started with an initial population of 1000 bacteria with a conventional growth rate, until the population size reached 20,000 bacteria. A solution with the tested metal was inoculated in the center of the colonies and diffused outwards. In the stationary state, the simulations were carried out with a virtually constant population of 2000 bacteria, which were completely submerged in the solution with the tested metal at a constant concentration. In both series of simulations, graphs with the number of bacteria expressing the corresponding chromoprotein over time were obtained. Furthermore, the set of simulations in the stationary state allowed validating the correct functioning of the designed biosensors, since such validations were carried out by immersing the colonies in three metal concentration conditions: at the activation threshold level, below the activation level, and no metal present in the solution [39].

Concerning the contributions of the present project, it should be noted that in previous works involving bacterial biosensors, most solutions were based on one of the following approaches: the use of hybrid designs involving bacteria and specialized hardware; *in vivo* designs implemented in molecular biology laboratories; and the use of sophisticated and expensive electronic equipment [40]. Therefore, the solution proposed in this article covers all *in silico* aspects related to design, modeling and simulation of exclusive whole-cell biosensors, and prepares the designs for their future *in vitro* implementation in the wet lab.

As future work, we propose the exploration of biocontainers for bacteria with an implementation of our designed biosensors to guarantee a safe deployment in the environment. In particular, we could work with a biocontainer based on hydrogel and elastomer, which could prevent bacteria from escaping the culture medium and spreading into the environment, but at the same time assuring the physical integrity of the bacteria and maintaining the correct functioning of the biosensors [41,42].

We also consider as future work to carry out a study on the operational feasibility and production cost of our biosensors designed *in silico* to be used in substitution of some of the methods currently in use for the detection of heavy metals in irrigation water for precision agriculture. Automation of the proposed development method, for instance by using artificial intelligence algorithms to help in the selection and assembly of the standardized biological parts, as well as in the simulation of the corresponding genetic circuit, should be investigated, thus linking the proposed methodology with the framework of *in vivo* artificial intelligence tools [43,44]. Since the research project focuses exclusively on *in silico* design, modeling, and simulation, laboratory experimentation is beyond the scope of this manuscript. However, the GenBank and FASTA output formats from the *in silico* process would enable laboratory experimentation by assembling each of the bioparts under the *BioBrick* standard (BBF RFC 10) [45].

Finally, considering that synthetic biology allows the programming of microorganisms so that they behave like small living computers, it could be advantageous to devote resources towards the study of new ways of making computing living devices that are less aggressive to the environment than other approaches. We envision that synthetic biology can help solve certain kind of problems without the need of elements such as integrated circuits, transistors, lithium batteries, capacitors, cables, and other hardware equipment, which can leave a persistent pollution footprint in our environment.

## 5 Conclusions

The use of software tools for the design of completely *in silico* bacterial biosensors represents a feasible alternative as a previous step to their *in vitro*/*in vivo* implementation in a molecular biology laboratory. These tools are constantly evolving, which demands the selection of the appropriate ones that can meet the requirements of the project at hand. In the present work we decided to use the software packages *iBioSim*, *TinkerCell*, and *gro*, as well the online *iGEM BioBricks* and *SynBioHub* databases. The set of tools and platforms chosen for the present project allows an integration of the whole process of design, modeling, and simulation, achieving full complementarity among the aforementioned tools to accomplish the research proposal.

Concerning the design of the three synthetic genetic circuits in *iBioSim*, this tool allowed not only importing the selected biological components into each circuit and their review with the *BioBricks* and *SynBioHub* databases, but also the validation of the complete genetic circuit. It was also possible to export the circuits in three standardized synthetic biology formats (FASTA, GenBank and SBOL), which expanded the possibility of using different software applications in which the proposed design can be replicated.

This work proposes a novel approach from a computer science point of view, not only from that of biology. We always prioritized the use of IT tools to carry out the entire process completely *in silico*. The proposed workflow allows professionals in the area of computer science to dabble in synthetic biology without the need for a laboratory or specialized knowledge in this area. At every stage we selected the software tool that we considered was the most appropriate to solve the problem at hand. To the best of our knowledge, no previous research had used the development pipeline *iBioSim*—>*TinkerCell*—>*gro* to perform the respective design, modeling, and simulation of synthetic biological circuits. We also found no previous report where a double validation of the standardized biological parts was used with the *iGEM BioBricks* and *SynBioHub* online databases. Finally, to make our results available to researchers who may want to carry out an *in vitro/in vivo* implementation of our designs, we used the SBOL international standard for synthetic biology. Our proposed methodology for biosensors can be used for the *in silico* development of other types of biological circuits before their *in vitro/in vivo* implementation.

However, our *in silico* approach has the drawback of depending on the availability of the biological parts that researchers working in laboratories publish in the *BioBricks* database, who in many cases may not be aware of the particular needs of researchers working on *in silico* designs. One possible solution to this problem could consist in escalating our proposed methodology in a way that researchers working with different approaches could receive direct feedback from each other with the goal of optimizing the *in silico* designs before passing to the *in vivo* implementation, in order to minimize time and cost.

## Data availability statement

All design (*iBioSim*), modeling (*TinkerCell*), and simulation (*gro*) files with complete genomes of each synthetic genetic circuit in FASTA and GenBank formats, as well as CSV files containing numerical results from various simulations, are available on GitHub. Additionally, high-definition and time-lapse versions of all 24 simulation videos are availabe on YouTube. The corresponding links are at the end of the Results section.

## CRediT authorship contribution statement

**Francisco Salcedo-Arancibia:** Writing – original draft, Visualization, Validation, Software, Methodology, Investigation, Conceptualization. **Martín Gutiérrez:** Writing – review & editing, Visualization, Validation, Supervision, Software, Methodology, Formal analysis, Conceptualization. **Arturo Chavoya:** Writing – review & editing, Validation, Supervision, Project administration, Methodology, Formal analysis, Conceptualization.

## Declaration of competing interest

We the authors of the paper intitled “Design, modeling, and *in silico* simulation of bacterial biosensors for detecting heavy metals in irrigation water for precision agriculture” have no competing interest to declare.
